# The C-terminus of nisin is important for the ABC transporter NisFEG to confer immunity in *Lactococcus lactis*

**DOI:** 10.1002/mbo3.205

**Published:** 2014-08-30

**Authors:** Zainab AlKhatib, Marcel Lagedroste, Julia Zaschke, Manuel Wagner, André Abts, Iris Fey, Diana Kleinschrodt, Sander H J Smits

**Affiliations:** 1 Institute of Biochemistry, Heinrich Heine University DuesseldorfUniversitaetsstrasse 1, 40225, Duesseldorf, Germany

**Keywords:** ABC transporter, immunity, lantibiotic, nisin, resistance

## Abstract

The lantibiotic nisin is a small 3.4 kDa antimicrobial peptide, which acts against Gram-positive bacteria in the nmol/L range. Nisin is produced and secreted by several *Lactococcus lactis* strains to ensure advantages against other bacteria in their habitat. Nisin contains five specific lanthionine rings of which the first two are important for Lipid II binding and the last two are crucial for the pore formation in the membrane. To gain immunity against nisin, the producing strain is expressing an ABC transporter called NisFEG, which expels nisin from the membrane. As a result six to eightfold more nisin is needed to affect the cells. The hydrolysis of ATP by NisFEG is required for this immunity as shown by a mutant, where the ATP hydrolysis is disrupted (NisF_H181A_EG). Furthermore, NisFEG recognizes the C-terminus of nisin, since deletion of the last six amino acids as well as of the last ring lowered the fold of immunity displayed by NisFEG.

## Introduction

Lantibiotics are small ribosomally synthesized peptides produced by numerous of gram-negative bacteria. After posttranslational modifications, lantibiotics are activated, upon the cleavage of the lantibiotic-specific presequence (or leader peptide), which is important for the modification machinery and subsequent secretion (Kuipers et al. 2004; Rink et al. 2005). These active lantibiotics are able to lyse mainly gram-positive bacteria as well as a limited number of gram-negative bacteria and act via different but distinct mechanisms (Wiedemann et al. 2001; Chatterjee et al. 2005b) targeting the membrane of the bacteria. This, however, also hold true for the membranes of the lantibiotic producer strains themselves. To circumvent this suicidal effect, lantibiotic producer strains express an immunity protein system (Kuipers et al. 1993; Siezen et al. 1996; Chatterjee et al. 2005b; Alkhatib et al. 2012). The genes encoding for the immunity system are present in all lantibiotic gene clusters found so far (Willey and van der Donk 2007). The two encoded proteins are called LanI, a membrane anchored lipoprotein, and LanFEG, an ATP-binding cassette (ABC) transporter localized in the cellular membrane. In most lantibiotic gene clusters both proteins are present although some exceptions are known (Chatterjee et al. 2005b; Alkhatib et al. 2012).

**Figure 1:**
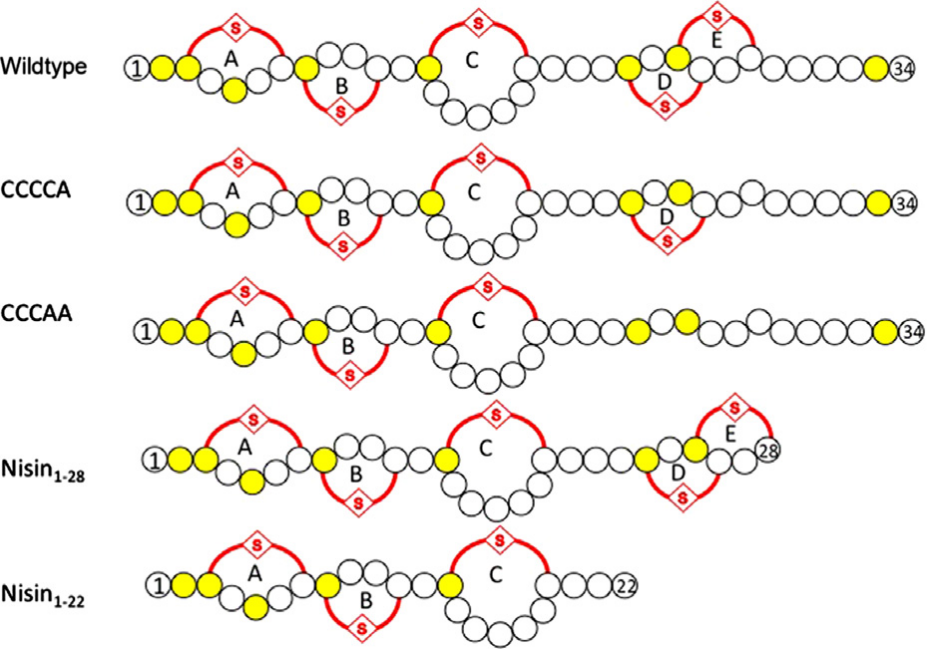
Nisin variants used in this study. Schematically shown is the wild-type nisin peptide structure as well as the variants CCCCA, CCCAA Nisin_1-28_ and Nisin_1-22_. Highlighted in yellow are the dehydrated residues and the lanthionine rings are highlighted with a red line. The lanthionine rings are numbered A–E.

Nisin is the best-known and most extensively studied lantibiotic, which is produced by some strains of *Lactococcus lactis* (*L. lactis*). Due to the high bactericidal activity in combination with the low toxicity in humans, nisin is used since decades as a natural preservative in the food industry (Delves-Broughton et al. 1996). Nisin is produced as an inactive precursor containing an N-terminal leader peptide that is cleaved after secretion by the subtilisin-like serine protease NisP (van der Meer et al. 1993). Active nisin is a small 3.4 kDa peptide, consisting of 34 amino acids and contains five lanthionine-based rings (ring A–E)(Fig.1). Here, the first three rings of nisin (ring A–C) are *N*-terminally located and are separated from the intertwined rings D and E (located at the C-terminus) by a flexible hinge region. The mode of action of nisin has been thoroughly studied, and it was shown that nisin attacks membranes both *in vivo* and *in vitro* (Ruhr and Sahl 1985; Van Den Hooven et al. 1996; Breukink et al. 1999). Nisin is inhibiting the cell-wall synthesis of mainly gram-positive bacteria by binding to the membrane using Lipid II as anchor point (Breukink et al. 1999). It has been shown that ring A and B of nisin are responsible for binding to Lipid II (Hsu et al. 2004), which is used as a “docking molecule” for nisin to protrude into the membrane by the hinge region as well as rings D and E to form a pore in the membrane (Brotz et al. 1998; Breukink et al. 1999; Wiedemann et al. 2001; van Heusden et al. 2002; Hasper et al. 2004). The presence of Lipid II is needed for the high activity of nisin, since in model membrane systems an increase in the pore formation activity by three orders of magnitude was observed when Lipid II was present. Furthermore, it has been shown that Lipid II is a constituent of the formed pore, which, when fully assembled, consists of four Lipid II and eight nisin molecules (Hasper et al. 2004).

The nisin producing *L. lactis* strains express two proteins involved in nisin immunity. The lipoprotein NisI and the ATP-binding cassette (ABC) transporter NisFEG, which when both are expressed result in immunity against a high level of nisin (Ra et al. 1996). Both immunity proteins seem to act cooperatively, since each of them displays only 8–20% of the full immunity when expressed alone (Ra et al. 1999; Stein et al. 2003). This cooperative mechanism between NisI and NisFEG is until now poorly understood.

ABC transporter like NisFEG, comprise one of the largest families of membrane proteins, found in all kingdoms of life. They transport a large variety of substrates ranging from small ions to large proteins (Hinsa et al. 2003) and can be subdivided into two major classes; i.e., export and import ABC transporter (Davidson et al. 2008). Generally these proteins consist of four domains, which are two hydrophobic transmembrane domains (TMD) and two hydrophilic cytosolic nucleotide-binding domains (NBD). The NBDs show high sequence similarity and contain characteristic sequence motifs like the Walker A, the Walker B, the H-loop, the C-loop or ABC signature motif (LSGGQ) (Schmitt and Tampe 2002), and the equally distinctive feature, the D-loop (van der Does and Tampe 2004; Higgins and Linton 2004; Zaitseva et al. 2006). The NBDs are involved in the binding and hydrolysis of ATP that provides energy for the transport of the substrate across the membrane.

In NisFEG, NisF represents the NBD, and the transmembrane domain comprises of the proteins NisE and NisG, forming a hetero-dimeric membrane complex, as observed for other ABC transporters (Siezen et al. 1996; Fetsch and Davidson 2002; Zaitseva et al. 2005). NisG is a 24 kDa integral membrane protein consisting of 214 amino acids with six predicted transmembrane spanning helices and NisE is a 28-kDa integral membrane protein containing a number of six predicted transmembrane helices as well (Alkhatib et al. 2012). Assembled as a complex, NisFEG forms a functional immunity ABC transporter with a proposed stoichiometry of NisF_2_EG (Chatterjee et al. 2005b). Deletion of either NisE or NisG abolished the immunity against the lantibiotic nisin completely, highlighting their equal importance for function (Siegers and Entian 1995). NisFEG has been shown to remove nisin from the membrane (Stein et al. 2003). Here, the authors showed that nisin can solely be found in the supernatant of the growth media, when NisFEG is expressed. Whereas some of the nisin molecules are membrane bound in cells lacking NisFEG. This leads to the conclusion that NisFEG exhibits a nisin expelling function (Stein et al. 2003).

Since the nisin-producing strains contain both immunity proteins, we used a homologous expression system, which allows the expression of only NisFEG in *L. lactis* by the *nisA* promotor. By determination of IC_50_ values of different *L. lactis* strains, we measured their immunity level against nisin. Beside the wildtyp (NZ9000NisFEG), we created a mutant, which is not able to hydrolyse ATP (NZ9000NisF_H181A_EG). This mutation revealed that without ATP hydrolysis no immunity is conferred and the level of nisin immunity is reduced to the level of the nisin-sensitive strain (NZ9000Erm). Furthermore, we investigated, which part of nisin potential can interact with NisFEG. We mutated nisin in such a way that (I) the last ring E and the last two rings D and E are not present, and (II) created truncation mutants of nisin, where the most last six and the last 14 amino acids are lacking. Hereby, we could show that NisFEG is indeed conferring immunity against nisin, and that the last ring as well as the most C-terminal located six amino acids of nisin are recognized by NisFEG and needed to fulfill its complete function.

## Material and Methods

### Cloning of pIL-SV

The *L. lactis*/*E. coli* shuttle vector pIL-SV was cloned using the In-Fusion HD cloning kit (Clontech) according to the manufacturers’ protocol. For this, the vector backbone of pIL3BTC (Rink et al. 2005) was amplified by PCR with the primers pIL-SV-for and pIL-SV-rev. The PCR, using the primers pUC19Inf-pIL-for and pUC19Inf-pIL-rev and the vector pUC19 as template, amplified a fragment with the coding region of the gene, which confers resistance to ampicillin in *E. coli*, and the pUC origin. In a third PCR the Promotor P_nisA_ was amplified applying the primers pIL-SV-P-for and pIL-SV-P-rev and pIL3BTC as template. The 15-bp overlap extension in the primers allowed the fusion of the three PCR fragments to the vector pIL-SV. Primers used in this study are given in Table1.

**Table 1:** Primers used in this study.

Name	Sequence (5′–3′)
pIL-SV-for	CAGCTTTCTTGTACAAAGTGGTGATGG
pIL-SV-rev	GGAGCTGTAATATAAAAACCTTCTTC
pUC19Inf-pIL-for	TTATATTACAGCTCCTCTTCCGCTTCCTCGCTCAC
pUC19Inf-pILrev	TATTGATCTTGGAGCGAAAGGGCCTCGTGATACGC
pIL-SV-P-for	GCTCCAAGATCAATAGAAACATTAAC
pIL-SV-P-rev	TGTACAAGAAAGCTGGCGGCCGCCTATTTGAGTGC
nisFEGNotI-for	CAAATAGGCGGCCGCATGCAGGTAAAAATTCAAAATCTTTCTAAAACATATAAAG
nisFEGSacI-rev	GAATTCGAGCTCCACAAGAAAAAATACTTTATCTAATCTTTTTTTTAG
NisF-H181A-for	GACAATCTTGATTTCTAGTGCTCAGTTGCACGAAATAAGTAAAG
NisF-H181A-rev	CTTTACTTATTTCGTGCAACTGAGCACTAGAAATCAAGATTGTC
CCCCA_for	CAGCAACTTGTCATGCTAGTATTCACGTAA G
CCCCA_rev	GCTTACCTGAATACTAGCATGACAAGTTGCTG
CCCAA_for	GGAGCTCTGATGGGTTGTAACATGAAA AC
CCCAA_rev	GTTTTCATGTTACAACCCATCAGAGCTCC
Nisin_1-22__for	GCTTACGTGAATTTAACAATGACAAGTTGC
Nisin_1-22__rev	GCAACTTGTCATTGTTAAATTCACGTAAGC

Shown are the primers used to create the pIL-SV-NisFEG and pIL-SV-NisF_H181A_EG expression plasmid, as well as the nisin mutants: CCCCA, CCCAA and the truncations Nisin_1-28_ and Nisin_1-22_.

### Cloning of pIL-SV-nisFEG and pIL-SV-nisF_H181A_EG

To construct the pIL-SV-nisFEG plasmid, the genes were amplified using isolated genomic DNA from *L. lactis* NZ9700 with the primers nisFEGNotI-for and nisFEGSacI-rev. The PCR-fragment and the vector pIL-SV were both hydrolyzed with *Not*I and *Sac*I and then ligated. The alanine substitution of the H181 residue by site-directed mutagenesis was performed by PCR using Pfu DNA polymerase, the template pIL-SV-nisFEG and a pair of oligonucleotides (NisF-H181A-for and NisF-H181-rev).

Both plasmids pIL-SV-nisFEG and pIL-SV-nisF_H181A_EG were verified by sequencing and then transformed into *L. lactis* NZ9000 by electroporation at 1 kV, 25 *μ*F, 5.0 msec, and the corresponding strain were termed NZ9000NisFEG and NZ9000NisF_H181A_EG. The used plasmids and bacterial strains are listed in Tables1 and 2.

**Table 2:** Strains used in this study.

Strain	Plasmid	Characteristics	Reference
NZ9000	–	nisRK+ and an empty plasmid	de Ruyter et al. (1996)
NZ9000NisFEG	pILSV-NisFEG	nisFEG	This work
NZ9000NisF_H181A_EG	pILSV-NisF_H181A_EG		This work
NZ9700	–	nisABTCIPRKEFG (Wild-type nisin producer)	Kuipers et al. (1993)

### Cloning of the prenisin variants

The mutants of nisin were introduced by site-directed mutagenesis using the pNZnisA vector, which contains an origin for *E. coli*, and therefore can be propagated in standard *E. coli* lab strains. The resulting plasmids were verified by sequencing. The used primers are listed in Table1 and the different variants are shown in Figure1. Four different variants have been constructed genetically, where the last or the last two cysteines where replaced by alanines, which when expressed results in active nisin containing only ring A–D or A–C, respectively. The resulting nisin variants are termed CCCCA and CCCAA, respectively (Fig.1). Furthermore, two truncation variants were used; one where the last six amino acids are missing termed Nisin_1-28_ (a kind gift of G. Moll Groningen), and one was newly cloned where the last 14 amino acids are missing termed Nisin_1-22_.

### Expression of NisFEG and NisF_H181A_EG in *L. lactis* NZ9000

The NZ9000NisFEG *and* NZ9000NisF_H181A_EG strains were grown in GM17 media supplemented with 5 *μ*g/mL chloramphenicol to an OD_600_ of 0.8. By the addition of nisin (final concentration of 1 ng/mL), the expression was induced and the culture was further grown overnight. After 15 h, the cells were diluted to an OD_600_ of 0.1 in fresh media supplemented with 5 *μ*g/mL chloramphenicol and with the inducer nisin (10 ng/mL).

### Purification of nisin

Nisin was basically purified as described in (Abts et al. 2011). In short, commercial available nisin powder (Sigma) was dissolved in 50 mmol/L lactic acid pH 3. The nisin solution was purified by using 5 mL HiTrap SP HP cation exchange column (GE Healthcare) pre-equilibrated with the same buffer. Nisin was eluted with 400 mmol/L NaCl and the elution fraction online monitored at a wavelength of 215 nm. Nisin containing fractions were precipitated by TCA and dried after washing it with cold acetone (Abts et al. 2011). Upon usage nisin was dissolved in 50 mmol/L lactic acid (pH 3) and the concentration of nisin was measured by using RP-HPLC (Abts et al. 2013).

### Expression and purification of prenisin variants

The expression of the nisin variants was performed as described in (Mavaro et al. 2011). The *L. lactis* NZ9000 strain harboring both pNZnisA containing the variants and pIL3BTC plasmids were used to express, modify and secret the prenisin variants in minimal medium (MM). The expression was induced by the addition of nisin to a final concentration of 5 ng/mL at OD_600_ of 0.4–0.5. The cells were harvested after overnight expression by centrifugation at 8000*g* for 30 min at 4°C.

The supernatant was diluted 1:1 with 50 mmol/L lactic acid, pH 3, and then applied on SP Sepharose column. Here, the buffer was changed to 50 mmol/L HEPES buffer pH 7, and the column was washed with eight column volumes with the same buffer. The prenisin variants were eluted with 50 mmol/L HEPES-NaOH pH 7.0, 1 mol/L NaCl and 10% (v/v) glycerol. The eluted prenisins were filtered through an Amicon Ultracentrifugal filter (30 kDa cut-off) to remove high-molecular weight proteins. The flow-through was concentrated afterwards using a 3 kDa cut-off Amicon Ultracentrifugal filter. The concentration of the different prenisins were determined by RP-HPLC (Abts et al. 2013).

### Expression and purification of NisP

The expression and purification of the serine protease NisP was previously described (Abts et al. 2013). In brief, the *L. lactis* strain NZ9000 harboring the plasmid pNG-NisP_8_His was grown in GM17 medium supplemented with 5 *μ*g/mL chloramphenicol overnight at 30°C. Cells were harvested, and transferred into minimal medium with a starting OD_600_ of 0.1 and 0.1 ng/mL of nisin was added to induce the expression of NisP.

After harvesting the cells, the pH of the supernatant was adjusted to pH 8.0 by the addition of 3 mol/L Tris-HCl (pH 10) and then applied to an IMAC HP column (GE Healthcare) preloaded with Co^2+^ and pre-equilibrated with low IMAC buffer (50 mmol/L HEPES-NaOH pH 8.0, 150 mmol/L NaCl). After washing with low IMAC buffer the NisP protein was eluted in one step with 50 mmol/L HEPES-NaOH, pH 8.0, 150 mmol/L NaCl, and 300 mmol/L imidazole. The elution fractions were concentrated by ultra-centrifugation (10 kDa MWCO) and the buffer was exchanged using a PD10 column (GE Healthcare) to 50 mmol/L HEPES-NaOH, pH 7.0, 150 mmol/L NaCl, and 10% glycerol. The resulting NisP protein was aliquoted and stored at −80°C until further usage.

### Activation of nisin and its variants

The different prenisin variants were incubated with NisP overnight at 30°C with a molar ratio of 1000:1. The activated nisin variants were used in all the assays described later. Prenisin and active forms of nisin variants were analyzed by RP-HPLC with a LiChrospher WP 300 RP-18 end-capped column using an acetonitrile/water solvent system as described (Abts et al. 2013) and further analyzed by Tricine-SDS-PAGE.

### Determination of the activity of nisin and its variants by IC_50_

The NZ9000, NZ9000NisFEG, and NZ9000NisF_H181A_EG strains were grown overnight in GM17 media supplemented with 5 *μ*g/mL chloramphenicol in the presence of 1 ng/mL nisin. The diluted cells (final OD_600_ was 0.1) were incubated with a serial dilution of nisin or its variants with 50 mmol/L lactic acid in a 96 well microtiter plate. The total volume in each well was 200 *μ*L, consisting of 50 *μ*L nisin and 150 *μ*L GM17 containing the corresponding *L. lactis* strain. The highest concentration of nisin used was adapted to the corresponding maximum immunity displayed by each strain.

The plate was incubated at 30°C. After 5 h, the optical density was measured at 620 nm via 96-plate reader BMG. The normalized optical density was plotted against the logarithm of the nisin concentration in order to calculate the IC_50_ of nisin and the data was evaluated using the following equation (1):
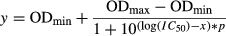
1

The IC_50_ value is the concentration of nisin were the growth of the *L. lactis* strain is inhibited by 50% as described in more detail earlier (Abts et al. 2011).

### SYTOx green nucleic acids binding

SYTOx green nucleic acids binding dye possesses a high-binding affinity towards nucleic acids. It enters cells only when they contain a pore in the plasma membrane and never crosses the intact membranes of living cells (Roth et al. 1997).

The cells of NZ9000, NZ9000NisFEG and NZ9000NisF_H181A_EG were grown overnight in GM17 supplemented with 5 *μ*g/mL chloramphenicol in presence of 1 ng/mL nisin. The next day, the overnight culture was diluted to an OD_600_ of 0.1 in fresh media supplemented with 5 *μ*g/mL chloramphenicol. The cultures were grown until an OD_600_ of 0.5 was reached. At this point the SYTOx green dye was added at a final concentration of 5 *μ*mol/L and incubated for 5 min according to the manual of the manufacturer (Invitrogen). The fluorescence signal, measured at an excitation and emission wavelength of 504 nm and 523 nm, respectively, was afterwards monitored for 200 sec to obtain a stable baseline. At 200 sec nisin was added and the fluorescence was monitored for 15 min.

## Results

### Activity of NisFEG against nisin

Active nisin was purified as previously described (Abts et al. 2011). The plasmid pILNisFEG or pILNisF_H181A_EG was transformed into *L. lactis* NZ9000 (termed NZ9000NisFEG and NZ9000NisF_H181A_EG). The expression of NisFEG was induced by the addition of externally added nisin (de Ruyter et al. 1996). To address the activity of nisin against the NZ9000Erm, NZ9000NisFEG and NZ9000NisF_H181A_EG strains, the IC_50_ value of nisin was determined for the different strains, which reflects the growth inhibition of the corresponding strain by 50%. The IC_50_ value was determined according to equation (1). The H181 in NisF was mutated into alanine, which was identified based on sequence comparison to be the H-loop, which is an essential sequence motif present in all ABC transporters. This mutation leads to NisFEG mutant, which can still bind ATP but ATP hydrolysis is prohibited.

**Figure 2:**
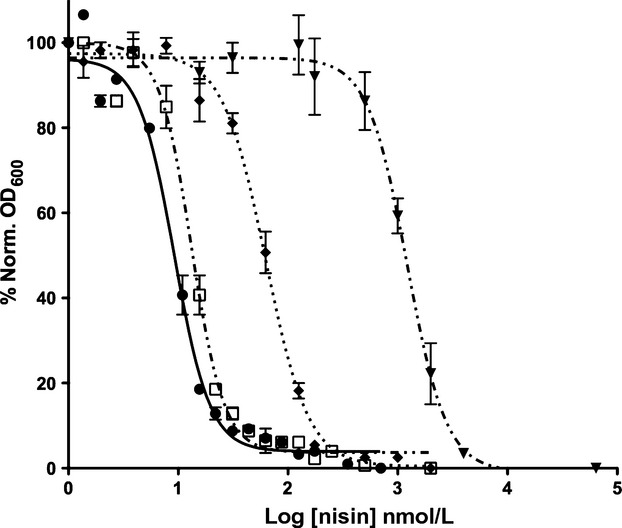
IC_50_ determination of nisin against different strains. Growth inhibition experiments were performed with nisin using the NZ9000Erm (●), NZ9000NisFEG (♦), NZ9000F_H181A_EG (open square) and NZ9700 strain (▼). Data was fitted and evaluated according to equation (1). Each experiment was performed at least in triplicates.

Nisin is highly active against the NZ9000Erm strain, which lacks the immunity system, as observed by the IC_50_ value of 9 ± 0.7 nmol/L (Fig.2 and Table3). The NZ9000NisFEG strain exhibited a seven to eightfold higher IC_50_ value of 59 ± 3.7 nmol/L (Fig.2 and Table3). This highlights that the ABC transporter NisFEG was able to confer immunity against nisin when expressed in *L. lactis*. The NZ9000NisF_H181A_EG strain displayed a lower IC_50_ value of 13 ± 1.2 nmol/L, which is similar to the value obtained for the NZ9000Erm strain. When using the *L. lactis* NZ9700 strain the IC_50_ value was 990 ± 54 nmol/L. This strain is a nisin producing *L. lactis* strain, which contains the full nisin operon, and thereby also the full immunity system consisting of NisI and NisFEG (Rauch and De Vos 1992). So the IC_50_ value determined for this strain reflects the activity of the full immunity system (100%). When NisFEG is expressed alone (strain NZ9000NisFEG) 6–8% of the immunity compared to the full immunity system is reached. This is in line with previous studies (Stein et al. 2003).

**Table 3:** IC_50_ values of nisin and its variants against the NZ9000Erm and NZ9000NisFEG strains.

	NZ9000Erm (nmol/L)	NZ9000NisFEG (nmol/L)	Fold of immunity
Nisin	9 ± 0.7	59 ± 3.7	6.5
CCCCA	74 ± 1.7	237 ± 32	3.6
CCCAA	182 ± 8	624 ± 87	3.4
Nisin_1-28_	177 ± 15	678 ± 78	3.8
Nisin_1-22_	224 ± 15	578 ± 63	2.5

Besides the IC_50_ values, also the fold of immunity against the nisin variants mediated by NisFEG is shown.

In summary, this shows that the ABC transporter NisFEG is able to confer a seven to eightfold immunity against nisin and that the NisF_H181A_EG is not showing any immunity (same immunity level as NZ9000Erm). This indicates that ATP hydrolysis is crucial for the function of the ABC transporter NisFEG.

### Pore formation of nisin in the membrane of NZ9000NisFEG cells

**Figure 3:**
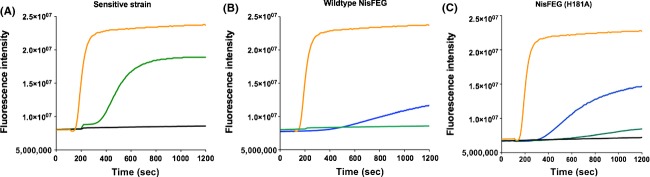
SYTOx green assay to visualize pore formation mediated by nisin. (A) NZ9000Erm strain (B) NZ9000NisFEG strain (C) NisF_H181A_EG were grown and incubated with the SYTOX green dye. The fluorescence signal was monitored online using a fluorolog (Horiba III). After a stable baseline was reached nisin was added and pore formation was monitored by measuring the increase in the fluorescence signal. Black curve: buffer control green curve 10 nmol/L nisin; blue curve 30 nmol/L nisin; orange curve 100 nmol/L nisin. The rapid increase in the fluorescence indicated pore formation.

Nisin is able to form pores in the membrane of gram-positive bacteria. This is mediated by the initial binding to Lipid II and subsequently reorientation of the C-terminal part of nisin into the membrane. This leads to membrane leakage and rapid cell death. We visualized this pore formation using a SYTOX green nucleic acid dye (Roth et al. 1997). In this assay, the dye enters the cells of *L. lactis* when pores are formed by nisin in the membrane. SYTOx enters the cells and binds DNA resulting in a rapid increase in the fluorescence signal, which can be monitored in real time. We monitored the pore forming action of nisin against the NZ9000Erm, NZ9000NisFEG, and NZ9000NisF_H181A_EG strains using three different nisin concentrations, 10, 30, and 100 nmol/L, respectively (Fig.3). As a control we added only the buffer, without nisin, which resulted in no increase in the fluorescence signal as observed by the black lines in Figure3A and C.

In line with the IC_50_ value for the sensitive strain (NZ9000Erm), pore formation was observed by the addition of 10 nmol/L nisin as indicated by the increase in the fluorescence signal (Fig.3A green curve). This increase was however not observed instantly, rather the effect started with a ∼200 sec delay. When adding a higher amount of nisin (e.g., 100 nmol/L) the increase in the fluorescence signal appeared instantly and reaches a stable plateau already after a couple of seconds (Fig.3A orange curve).

The NZ9000NisFEG strain was not affected when 10 nmol/L nisin was added as no increase in the fluorescence signal was observed (Fig.3B green curve). When adding 30 nmol/L nisin to the NZ9000NisFEG strain, which corresponds to 50% of the determined IC_50_ value, also no significant increase was observed. Here, however a small linear increase was visible, indicating that some of the cells were lysing (Fig.3B blue line). When adding a nisin concentration above the IC_50_ value, e.g., 100 nmol/L to the NZ9000NisFEG strain, the fluorescence signal was increasing rapidly, indicating that NisFEG is not able to confer immunity at this nisin concentration. The NZ9000NisF_H181A_EG strain showed almost no effect upon the addition of 10 nmol/L of nisin. This concentration was below the IC_50_ value determined for this strain (see above). For the NZ9000NisF_H181A_EG strain an increase in the fluorescence signal is observed at 30 nmol/L nisin (Fig.3C blue curve). Indicating that this strain is not able to counteract this concentration of nisin. Here, similar to the observed curve for the NZ9000Erm strain with 10 nmol/L nisin, the increase in the fluorescence signal occurs after a delay of 200 sec. Upon the addition of 100 nmol/L nisin also the NZ9000NisF_H181A_EG strain displayed a rapid almost instant increase in the fluorescence signal (Fig.3C orange curve).

Altogether this shows that NisFEG confers immunity against nisin when expressed in *L. lactis*, up to 60 nmol/L by preventing pore formation. At nisin concentrations above the IC_50_ value, NisFEG cannot protect the cells against nisin anymore, as a rapid increase in the fluorescence signal was observed. Hence, nisin is able to form pores, at this elevated concentration. In this assay again the NisF_H181A_EG mutant is not able to confer immunity due to the lack of ATP hydrolysis.

### Purification and activity of nisin variants

Nisin contains five lanthionine rings, which are crucial for its antimicrobial activity. Whereas, the first two rings are important for docking onto Lipid II, the last two are important for the pore formation in the *L. lactis* membrane (Wiedemann et al. 2001; Hsu et al. 2004). We constructed variants of nisin were genetically the last or the last two cysteines were replaced by alanines, resulting in active nisin variants containing ring A–D or ring A–C, respectively (Fig.1, primers are listed in Table1). An overview of the different variants is schematically shown in Figure1. After successful introduction of the mutations, the resulting plasmids were transformed into *L. lactis* together with the pIL3BTC plasmid encoding for the modification and secretion machinery. This dual plasmid system has been previously used to introduce successfully mutations in the leader peptide (de Ruyter et al. 1996; Abts et al. 2013). The resulting nisin variants are termed CCCCA and CCCAA, respectively (Fig.1). Furthermore, we constructed two truncation variants; one where the last six amino acids are missing termed Nisin_1-28_. Important to note here, is that the five lanthionine rings, A–E are still installed (Fig.1). The Nisin_1-22_ variant contained a stop codon at position 22 and its expressed prenisin variant contained the first three lanthionine rings A–C, but lacks the C-terminus (Fig.1). The expression and purification was performed as described in Material and Methods.

**Figure 4:**
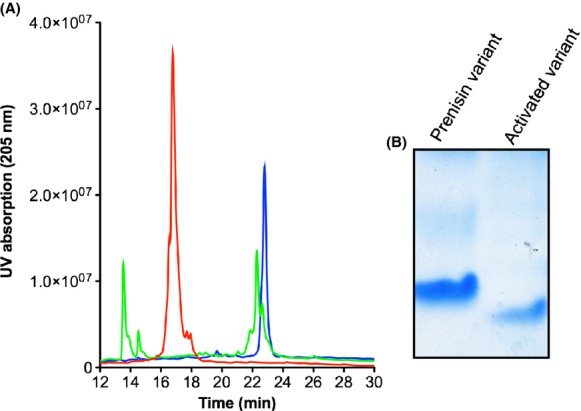
Cleavage reaction of prenisin variants analyzed by RP-HPLC: (A) RP-HPLC profile following the cleavage reaction of NisP with the modified CCCCA precursor peptide. The modified precursor CCCCA peptide (red) was digested with NisP and analyzed by RP-HPLC. The digested sample (green) showed two peaks. One at an elution time of 13.5 min corresponding to the leader peptide, which was confirmed by mass spectrometric analysis. The second peak eluted at 22 min, which run similar as the active nisin (blue) which was used as control. (B) SDS-PAGE analysis of the activated nisin variants. Exemplary shown is the activation of the CCCCA variant. Left lane: prenisin form of the CCCCA variants. Right lane: activated nisin variant after NisP treatment and HPLC analysis.

After secretion of the prenisin variants, the supernatant was applied onto a cation exchange chromatography (cIEX) and eluted using high salt buffer (see Material and Methods). To remove higher molecular weight species the samples were spawn through a 30-kDa concentrator were the prenisin variants can be collected in the flow trough. Since these variants are inactive with the leader sequence attached we cleaved off the leader sequence by incubation with purified NisP in a molar ratio of 1000:1. We analyzed the cleavage reaction via RP-HPLC (Fig.4A). The prenisin variants eluted at a retention time of 17.2 min (Fig.4A red line). After cleavage the peak diminished and two new peaks appeared. The first peak eluted at 13.9 min and the second peak appeared at a retention time of 22.2 min (Fig.4A green line). As a control active nisin purified from the commercial powder was injected onto the RP-HPLC and elutes at a retention time of 22.5 min (Fig.4A blue line). The peaks were collected and analyzed by mass spectrometric analysis to verify the identity of the peptides eluting and revealed that the cleavage efficiency of the prenisin variants reached nearly 100%. The analysis of the first peak revealed that this contained a peptide, which corresponds to the leader sequence, whereas the second peak contained the corresponding nisin variant. The peaks containing these activated nisin variants were used to determine the antimicrobial activity against the different strains.

The nisin variants were >90% pure as judged by Tricine-SDS-PAGE analysis (Fig.4B). The exact concentration for each variant as well as wild-type nisin was determined via RP-HPLC analysis, by the integration of the corresponding peaks as described before (Abts et al. 2013).

**Figure 5:**
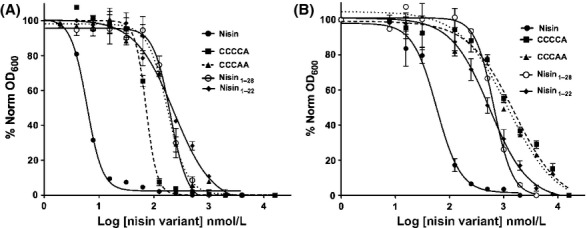
IC_50_ determination of the CCCCA, CCCAA, Nisin_1-28_ and Nisin_1-22_ against the (A) NZ9000Erm and (B) NZ9000NisFEG strains. Growth inhibition experiments were performed with the nisin variant using the NZ9000Erm and NZ9000NisFEG. Data was fitted and evaluated according to equation (1). Each experiment was performed at least in triplicates.

The nisin CCCCA and CCCAA variants, displayed IC_50_ values of 74 ± 1.7 and 182 ± 8 nmol/L, against the sensitive NZ9000Erm strain, respectively (Fig.5A and Table3). This resembles an eight to ninefold increase of the IC_50_ value but a 20-fold reduction in the antimicrobial activity (compared to wild-type nisin). This is consistent with previous studies highlighting that the C-terminal part of nisin is crucial for its high activity in the low nmol/L range (Rink et al. 2007).

Similarly, the truncated mutations also showed a significantly reduced activity. The Nisin_1-28_ variant lacking the last six amino acids showed an IC_50_ of 177 ± 15 nmol/L (20-fold), whereas the Nisin_1-22_ variant displayed an IC_50_ of 224 ± 15 nmol/L (25-fold). This shows that not only the last two rings are important for the activity of nisin, but also that the last six amino acids play a crucial role, since their deletion lowered the activity 12-fold (Fig.5A and Table3).

### Substrate specificity of NisFEG

NisFEG confers seven to eightfold immunity as observed when comparing the IC_50_ value of wild-type nisin against the NZ9000Erm and the NZ9000NisFEG strain (see above). This seven to eightfold of immunity can now be used to detect whether the nisin mutants have an effect on the activity of NisFEG. A similar increase of the IC_50_ of the nisin variants would indicate that NisFEG still exhibits 100% activity. If the factor decreases between the IC_50_ values, the nisin variants are not recognized or not as efficiently expelled from the membrane by NisFEG.

For the CCCCA, we observed a 3.2-fold increase of the IC_50_ value (237 ± 32 nmol/L; Fig.5B) when comparing the IC_50_ values for this variant against the NZ9000Erm strain and NZ9000NisFEG strain (Fig.5B and Table3). Similarly, the nisin variant CCCAA displayed a 3.4-fold immunity (IC_50_ value of 624 ± 87 nmol/L for the NZ9000NisFEG strain compared to 182 ± 8 nmol/L of the NZ9000Erm strain [Fig.5B]). This shows that the deletion of ring E lowered the activity of NisFEG by 50%. The simultaneously deletion of both rings D and E however, did not result in a further reduction in the immunity mediated by NisFEG. This implies that that ring E is more important than ring D for the activity of NisFEG.

The nisin truncation variants Nisin_1-28_ and Nisin_1-22_ displayed IC_50_ values of 678 ± 78 and 578 ± 63 nmol/L, against the NZ9000NisFEG strain, respectively. This represents a 3.8-and 2.5-fold increase compared to the values obtained for the NZ9000Erm strain (Fig.5B and Table3). This shows that NisFEG is not only able to confer immunity for 100% when nisin is lacking its C-terminus, especially the last six amino acids. The deletion of the last six amino acids reduced the activity of NisFEG to 60%. The Nisin_1-22_ variant reduces the activity of NisFEG to 33%. Taken together the C-terminal part of nisin is recognized by NisFEG.

## Discussion

Lantibiotics are small posttranslationally modified peptides, which display a high antimicrobial activity against numerous gram-positive bacteria. The best-characterized lantibiotic is nisin, which is produced by several *L. lactis* strains (Piper et al. 2011). This, 3.4 kDa antimicrobial peptide contains five lanthionine rings (ring A–E), specifically introduced by two enzymes (Koponen et al. 2002). These lanthionine rings are crucial for the high level of antimicrobial activity as well as protection against proteolytic degradation (Chatterjee et al. 2005a). To confer immunity against nisin, the nisin producer *L. lactis* strain co-expresses the membrane-associated protein systems NisI and NisFEG (Kuipers et al. 1993). The expression of these genes is regulated by a two-component system, consisting of NisR and NisK, which is able to sense the external nisin concentration (van der Meer et al. 1993; Ra et al. 1996).

The proteins of the immunity system, NisI and NisFEG, act cooperatively, since full immunity is only observed when both proteins are present simultaneously (Kuipers et al. 1993; Stein et al. 2003). This was shown in *L. lactis* itself by knockout studies (Ra et al. 1996) as well as by heterologous expression in *Bacillus subtilis* (Stein et al. 2003). A strain with the full immunity system gained immunity against nisin even at high concentrations, whereas the single expression of one of the genes reduced this nisin immunity drastically (Stein et al. 2003). Recently, a function of NisI has been described. NisI inhibits nisin is able to inhibit nisin mediated pore formation up to a concentration of 1 *μ*mol/L nisin (AlKhatib et al. 2014). This activity is mediated by the C-terminus of NisI, suggested to be involved in binding Lipid II. The immunity conferred by NisI seems to be directly targeted towards the nisin–Lipid II interaction.

NisFEG is however involved in expelling nisin directly from the membrane prior to pore formation. So both immunity protein systems seems to be active on different mode of actions of nisin. Since the pore formation of nisin is mediated by the C-terminal located rings D and E, we focused on these rings to characterize the function of NisFEG.

In this study, we showed that NisFEG, when homologously expressed in *L. lactis* is conferring a seven to eightfold immunity, as reflected by the increase of the IC_50_ value (Table3). Furthermore, we observed that NisFEG is protecting the membrane from pore formation by nisin up to a 60 nmol/L concentration. However at nisin concentrations above this IC_50_ value, nisin is able to penetrate the cells and pore formation can be observed again. These results are in line with studies using NisFEG expressed *B. subtilis*. Here it was shown that the strains expressing NisFEG expel nisin from the membrane back into the medium (Stein et al. 2003). The epidermin immunity ABC transporter EpiFEG from *S. epidermidis* also exhibits such an expelling function to confer immunity (Otto et al. 1998).

NisFEG can only provide immunity up to a certain level and the velocity of the ABC transporter dictates maximum nisin concentration that can be expelled. At nisin concentration above 60 nmol/L, NisFEG cannot remove all nisin molecules anymore and some nisin molecules will penetrate the membrane and pores are formed leading to cellular leakage and consequently cell death. This is observed in our SYTOx green assay where at concentration above the IC_50_ value pore formation is observed.

ABC transporters, like NisFEG, contain specific sequence motifs, which are essential for the binding and/or hydrolysis of ATP. These motifs, Walker A and B, the C-loop and D-loop form the hall marks of this protein superfamily and are localized in the nucleotide-binding domain (NisF). By sequence alignment we identified H181 in NisF to be the H-loop. Mutations of the H-loop have been found to abolish ATP hydrolysis completely in other ABC transporter systems. For example in the nucleotide-binding domain of the Type 1 secretion system of *E. coli* the introduction of the H662A mutation yielded protein, which is still able to bind ATP with similar affinities. But ATP hydrolysis is disrupted and therefore the protein is not active. We expressed the H181A mutant and observed that the corresponding NZ9000NisF_H181A_EG strain was not able to display any significantly nisin immunity anymore. The IC_50_ value of the NZ9000NisF_H181A_EG strain (13 nmol/L) is similar to the value of the nisin sensitive strain NZ9000Erm (9 nmol/L). This highlights ATP hydrolysis is crucial for the activity of NisFEG.

Nisin itself has several modes of action: binding to Lipid II, which results in growth inhibition and displacement of Lipid II, which also inhibits the growth (Wiedemann et al. 2001; Hasper et al. 2006). Both these mechanism rely on the binding of nisin to Lipid II, which is mediated by the first two lanthionine rings. The high activity of nisin, however is achieved by pore formation. The formation of these pores require a specific transmembrane orientation of the C-terminal part of nisin (van Heusden et al. 2002), which is possible due to a flexible hinge region (nisin residues 20−22; in between ring A–C and rings D and E). Since NisFEG is expelling nisin from the membrane (Stein et al. 2003), it seems plausible that NisFEG is recognizing the C-terminal part of nisin. We created four different variants of nisin to investigate this, two of them lack ring E or both rings D and E, termed CCCCA and CCCAA, respectively. Two other variants (termed Nisin_1-28_ and Nisin_1-22_) were truncations of nisin, where the last six amino acids or the last 12 amino acids were missing. In the Nisin_1-28_ truncation all rings are still present, whereas in the Nisin_1-22_ only ring A–C and the linker region are left.

The CCCCA and CCCAA nisin variants still display some antimicrobial activity though lower as the wild-type nisin. Against the NZ9000Erm strain the CCCCA mutant displayed a 8-fold (74 ± 1.7 nmol/L) and for the CCCAA mutant an almost 20-fold (182 ± 8 nmol/L) lower activity. This novel findings illuminates that rings D and E are crucial for the activity of nisin as suggested by other (Hsu et al. 2004). Also the truncated mutants displayed a lower IC_50_ against the NZ9000Erm strain. Here, the activity dropped for Nisin_1-28_ by a factor of 20 and for Nisin_1-22_ a 25-fold reduction was observed. Similar to previous studies where the C-terminal deletion of nisin leads to a 16–100-fold reduction of the activity (Chan et al. 1996; Sun et al. 2009). This shows that, not only the lanthionine rings of nisin are important for the activity, but also the most C-terminal residues play an important role.

We used the variants of nisin to analyze the fold of immunity mediated by NisFEG. This allowed us to investigate whether NisFEG is still able to expel the nisin variants from the membrane with similar or different efficiencies.

After performing the same experiments with the nisin variants, we observed that the fold of immunity mediated by NisFEG reduced by 50% when deleting ring E. The same reduction was observed when both rings D and E were not present. This shows that ring E is most important and that the loss of ring D has no impact on the observed immunity. The fold of immunity decreased also 50% for the nisin truncation variant Nisin_1-28_ (3.4-fold). Here, all five rings are still present. So, the missing last six C-terminal six amino acids and ring E are equally important for the activity of NisFEG.

Our nisin variants showed that the most C-terminal part of nisin is of special need to be expelled by NisFEG. When these amino acids are missing as in the Nisin_1-28_ variant, the ABC transporter is still able to confer some immunity, however at a lower level. This suggests that, besides this C-terminus, also other parts are needed for the immune activity of NisFEG. Presumably, also ring A and B play a role as they bind Lipid II, which is needed prior to pore formation. The activity of NisFEG is therefore depending on the C-terminus of nisin, which correlated nicely with the fact that this part of nisin is crucial for the pore formation mode of action.
